# Platypus globin genes and flanking loci suggest a new insertional model for beta-globin evolution in birds and mammals

**DOI:** 10.1186/1741-7007-6-34

**Published:** 2008-07-25

**Authors:** Vidushi S Patel, Steven JB Cooper, Janine E Deakin, Bob Fulton, Tina Graves, Wesley C Warren, Richard K Wilson, Jennifer AM Graves

**Affiliations:** 1 The ARC Centre for Kangaroo Genomics, Research School of Biological Sciences, The Australian National University, Canberra, ACT 2601, Australia; 2 Australian Centre for Evolutionary Biology and Biodiversity, The University of Adelaide, Adelaide, SA 5005, Australia; 3 Evolutionary Biology Unit, South Australian Museum, Adelaide, SA 5000, Australia; 4 Genome Sequencing Center, Washington University School of Medicine, St Louis, Missouri 63108, USA

## Abstract

**Background:**

Vertebrate alpha (α)- and beta (β)-globin gene families exemplify the way in which genomes evolve to produce functional complexity. From tandem duplication of a single globin locus, the α- and β-globin clusters expanded, and then were separated onto different chromosomes. The previous finding of a fossil β-globin gene (ω) in the marsupial α-cluster, however, suggested that duplication of the α-β cluster onto two chromosomes, followed by lineage-specific gene loss and duplication, produced paralogous α- and β-globin clusters in birds and mammals. Here we analyse genomic data from an egg-laying monotreme mammal, the platypus (*Ornithorhynchus anatinus*), to explore haemoglobin evolution at the stem of the mammalian radiation.

**Results:**

The platypus α-globin cluster (chromosome 21) contains embryonic and adult α- globin genes, a β-like ω-globin gene, and the *GBY *globin gene with homology to cytoglobin, arranged as 5'-ζ-ζ'-α^D^-α^3^-α^2^-α^1^-ω-*GBY*-3'. The platypus β-globin cluster (chromosome 2) contains single embryonic and adult globin genes arranged as 5'-ε-β-3'. Surprisingly, all of these globin genes were expressed in some adult tissues. Comparison of flanking sequences revealed that all jawed vertebrate α-globin clusters are flanked by *MPG*-*C16orf35 *and *LUC7L*, whereas all bird and mammal β-globin clusters are embedded in olfactory genes. Thus, the mammalian α- and β-globin clusters are orthologous to the bird α- and β-globin clusters respectively.

**Conclusion:**

We propose that α- and β-globin clusters evolved from an ancient *MPG-C16orf35-α*-β-*GBY-LUC7L *arrangement 410 million years ago. A copy of the original β (represented by ω in marsupials and monotremes) was inserted into an array of olfactory genes before the amniote radiation (>315 million years ago), then duplicated and diverged to form orthologous clusters of β-globin genes with different expression profiles in different lineages.

## Background

The evolution of the vertebrate globin superfamily has been extensively studied for many decades by comparing the structure and function of members of the gene families. These are principally haemoglobin, myoglobin, cytoglobin and neuroglobin and, more recently, globin X (in fish and amphibians [1]) and globin Y (specific to amphibians [2]).

Haemoglobin genes (alpha- and beta-globin) are of particular interest because of their critical role in oxygen transportation from the respiratory surfaces to the inner organs, and because of the dire effects of mutations in human globin genes that cause haemoglobinopathies [3]. The genes contained in the alpha (α)- and beta (β)-globin clusters are expressed at different stages of development and in different tissues. Together, gene products from both clusters form the functional tetrameric haemoglobin molecules needed to fulfil oxygen requirements.

The evolutionary history of α- and β-globin genes can be traced back to the common ancestors of fish, amphibians and amniotes (reptiles, birds and mammals), by comparing gene structure and composition of α- and β-globin clusters across vertebrates. In the amphibians *Xenopus laevis *and *X. tropicalis*, α- and β-globin genes are tightly juxtaposed as 5'-α-β-3' [2,4-6]. In the Antarctic notothenioid fish (*Notothenia coriiceps, N. angustata, Trematomus hansoni, T. pennellii*), there is also a single 5'-α-β-3' locus [7], although in pufferfish (*Fugu rubripes*) there are two globin clusters (one with α-globin genes and the other with both α- and β-globin genes), which are located on different chromosomes [8].

In amniotes, α- and β-globin clusters are located on different chromosomes. It was proposed that the ancestral α- and β-globin genes were located together in the common ancestor of amniotes, as they are in fish and amphibians, but became separated, either by chromosome fission or translocation between α- and β-genes, or by chromosome/genome or *in trans *duplication and gene loss [5].

Further duplications then occurred in amniote lineages. The ancestral α-globin gene is thought to have duplicated twice before the divergence of the bird-mammalian lineages, to produce progenitors of embryonic globin genes π/ζ, and adult α^D ^and α^A^, all of which are present in birds (for example, the chicken *Gallus gallus*) [9-11] and mammals [12,13]. The order and timing of these duplications is still debated, as is their origin: for instance, α^D ^may have evolved by duplication either of adult α^A ^(see [12]), or of an embryonic α-like gene [14]. After the avian and mammalian lineages diverged, there were further tandem duplications of the π/ζ and α^A ^lineages to produce more complex marsupial and eutherian ('placental') mammalian α-globin clusters, 5'-ζ-ψζ'-α^D^-ψα^3^-α^2^-α^1^-θ-3' (see [12,15-18]). The timing of these duplication events is also uncertain, because we do not know whether these seven α-like globin genes all existed at the stem of the mammalian radiation.

As for many other gene families [19], comparisons of globin genes between distantly related mammals have provided unique insight into the evolution and function of the mammalian globins. Marsupials diverged from eutherian mammals about 148 million years ago (MYA), and mammalian Subclass Theria that contains these groups diverged from monotremes (Subclass Prototheria) about 166 MYA [20], so comparisons between these major mammal groups provide depth for evolutionary comparisons. Monotremes retain many anatomical and developmental features shared with birds and reptiles. Their small genome, too, and disjunct chromosome size classes are reminiscent of reptile genomes, and the 10 sex chromosomes in a karyotype of 52 chromosomes is unique among mammals [21-23]. Their importance for comparative studies is now increasingly recognised after the sequencing of the genome of a monotreme, *Ornithorhynchus anatinus *(platypus), to a depth of six to eight times by the Washington University Genome Centre, St Louis [24].

Indeed, studies of marsupial globins have clarified the timing of some of the duplications. The finding of single ε- (embryonic) and β-globin (adult) genes together in the marsupial β-globin cluster indicated that a two-gene cluster (ε-β) was present in the common therian ancestor [25-28]. Genes in the cluster were further duplicated to produce the ancestral eutherian β-globin cluster of 5'-ε-γ-η-δ-β-3' (see [29-32]), which then underwent further tandem duplication events. In contrast, the bird (*G. gallus*) β-like globin genes (ε-β^H^-β^A^-ρ) show very little homology to the mammalian β-like globin genes [33,34].

The discovery of a β-like globin gene (ω *-*globin) adjacent (3') to the α-globin cluster in marsupials led to a re-interpretation of globin evolution in birds and mammals [35,36]. Comparative sequence and phylogenetic analysis suggested that the ω-globin gene was more closely related to bird β-like globin genes than to other mammalian β-like globin genes. The specific function of the ω-globin gene is not yet known, but it is expressed just before birth and in the early stages of pouch young development [37]. In addition, the ω-globin product binds to α-like globin chains to form functional haemoglobin, so it is likely to be involved in oxygen transportation [35-37].

**Figure 1:**
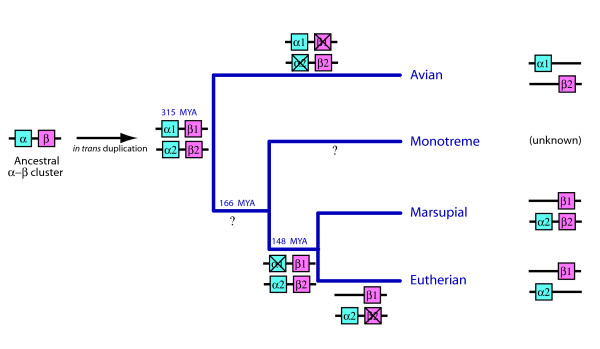
**Current proposed model for the evolution of α- and β-globin clusters from paralogous clusters in different lineages**. The unlinked α- and β-globin clusters in birds and mammals evolved from an ancient *in trans *duplication of the ancestral linked α-β cluster, followed by differential gene silencing (marked with X). This resulted in bird β-like globin genes (β2) orthologous to the marsupial ω-globin gene (β2 beside the α-globin cluster) but paralogous to mammalian β-like globin genes (β1). Adapted from Wheeler et al. [36].

This finding of a remnant β-like globin gene (ω *-*globin) beside the α-globin cluster in marsupials [35,36] provided some support for the alternative hypothesis [5] that the α- and β-globin clusters in birds and mammals arose by *in trans *duplication of a chromosomal region, rather than simply by separation of the ancestral α-β globin cluster by chromosome fission or translocation. Wheeler et al. [35,36] proposed that before the divergence of birds and mammals (>315 MYA), the chromosome region bearing the ancestral α-β clusters duplicated to form two clusters (α1-β1 and α2-β2) on different chromosomes, and their contents diverged independently in mammals and birds by silencing of some genes within each cluster (Figure 1). To account for the apparent orthology of the marsupial ω-globin gene and bird β-like globin genes, Wheeler et al. [35,36] suggested that the α1 and β2 were silenced in the eutherian lineage, but β2 was retained in marsupials as the ω-globin. In contrast, α2 and β1 were silenced in the bird lineage (Figure 1). On this hypothesis, then, both the α clusters and the β clusters of birds and mammals are paralogous (that is, evolved independently from ancient duplicates in an amniote ancestor) rather than orthologous (that is, diverged from the same ancestral cluster in an amniote ancestor).

This paralogy hypothesis (which rests on the rather weak orthology between the chicken β and marsupial ω), as well as the dates and types of other duplications, could be further tested by studying globin genes of monotreme mammals, and using comparative data to infer the ancestral globin gene arrangement of a mammal ancestor 166 MYA. The availability of platypus genomic sequences now provides an efficient way to discover all of the globin genes and regulatory signals, and to understand their function and evolution. Studies of globin genes in monotremes are also interesting because the specialized features and lifestyle of these unique mammals may have given rise to special adaptations of globin genes to fulfil unusual oxygen requirements. These features include the need for oxygen by diffusion through the egg membrane to the embryo after birth and the physiological response to hypoxic conditions during hibernation, burrowing and diving [38-40].

Little is known about monotreme α- and β-globin families. More than 30 years ago, studies of adult blood revealed a single adult α and β globin protein in the platypus [41,42] and echidna (*Tachyglossus aculeatus *[43,44]). Lee et al. [28] later isolated an adult β-globin gene in the echidna that encoded a polypeptide identical to the previously isolated echidna β-globin [44]. To date, there is no evidence of any monotreme embryonic ζ- or ε-globin genes.

We used platypus genomic sequences from bacterial artificial chromosomes (BACs) to characterise the α- and β-globin gene families of the platypus and investigate their molecular evolution. In particular, we searched for embryonic and ω-globin genes and any novel globin genes that might fulfil the requirements for oxygen transport under hypoxic conditions. We investigated the genome context in order to infer the structure and origin of the ancestral α- and β-globin clusters at the stem of the mammalian radiation. Our results strongly support the hypothesis that the mammalian α- and β-globin clusters are orthologous to the avian α- and β-globin clusters, respectively, and that the β cluster evolved by transposition of a copy of the beta-like ω-globin gene in an amniote ancestor.

## Results

### Identification of BAC clones containing the α- and β-globin clusters

The draft sequence assembly of platypus [24] is readily available on the University of California Santa Cruz (UCSC) Genome Browser [45]. However, currently the assembly is incomplete for the α- and β-globin clusters, as individual globin genes appear on different contigs. There are also sequences of the platypus BAC clones available in NCBI GenBank that are not yet annotated and assembled, nor is part of the platypus genome assembly. Two of these are Oa_Bb-2L7 [GenBank:AC195438] and Oa_Bb-131M24 [AC203513], which were identified from the Encyclopaedia of DNA Elements Project to contain parts of the α-globin cluster (see Methods). The BAC clone Oa_Bb-484F22 [GenBank: AC192436] containing the β-globin cluster was obtained by screening a male platypus BAC library (Clemson University Genomic Institute, USA) and was subsequently fully sequenced and assembled by the Washington University Genome Sequencing Centre (St Louis, USA). These sequences were therefore used in this study to characterise the whole α- and β-globin clusters in the platypus.

**Figure 2:**
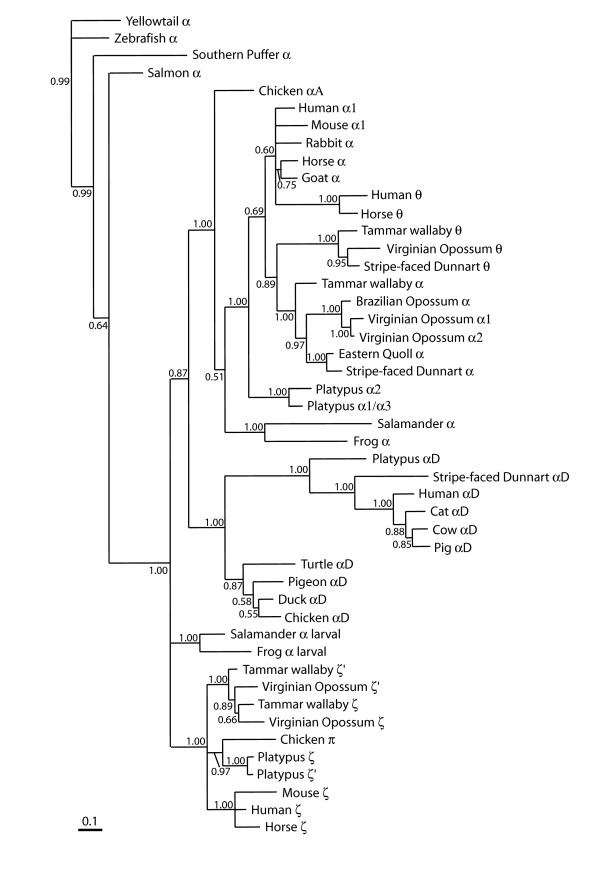
**Evolutionary relationships among vertebrate α-like globin genes using a 50% majority rule consensus phylogram from an analysis using Bayesian Inference**. The tree was constructed using mixed models of evolution for each codon position (see methods) and estimated base frequencies in an unlinked analysis using MrBayes (v. 3.1.2). Numbers adjacent to branches refer to % posterior probabilities. GenBank accession numbers for sequences are: Virginian Opossum (*Didelphis virginiana*) ζ^1^, ζ^2^, α^1^, α^2^, θ [AC139599.2, AC148752.1]; Stripe-faced Dunnart (*Sminthopsis macroura*) α^D^, α^2^, θ [AC146781]; Brazilian Opossum (*Monodelphis domestica*) α [TI# 453585430]; Tammar wallaby (*Macropus eugenii*) θ [AY459590], α [AY459589]; ζ [AY789121], ζ' [AY789122]; Horse (*Equus caballus*) θ (ψ α) [Y00284], α^1 ^[M17902], ζ [X07051]; pig (*Sus scrofa*) α^D ^[AC145444]; cat (*Felis catus*) α^D ^[AC130194]; cow (*Bos taurus*) α^D ^[AC150547]; Goat (*Capra hircus*) α [J00043]; Human (*Homo sapiens*) α1 [V00491], θ [X06482], ζ [NM_005332]; *mu*/α^D ^chain [AY698022]; Mouse (*Mus muscularis*) α^1 ^[NM_008218], ζ [X62302]; Rabbit (*Oryctolagus cuniculus*)α [X04751]; Eastern Quoll (*Dasyurus viverrinus*) α [M14567]; Chicken (*Gallus gallus) *α^A^, π, α^D ^[AF098919]; Duck (*Cairina moschata*) α^D ^[X01831]; Pigeon (*Columba livia*) α^D ^[AB001981]; Turtle (*Geochelone nigra*) α^D ^[SEG# AB1165195]; Zebrafish (*Danio rerio*) α^1 ^[NM_131257]; Salamander (*Hynobius retardatus*) larval α [AB034756]; Salamander (*Pleurodeles waltlii*) α [*M*13365]; Frog (*Xenopus laevis*) α *I *[X14259], larval (tadpole) α T5 [X02798]; Yellowtail (*Seriola quinqueradiata*) α^A ^[AB034639]; Salmon (*Salmo salar*) α [X97289]; Southern Puffer (*Sphoeroides nephelus*) α^2 ^[AY016023]; Platypus (*Ornithorhynchus anatinus*) ζ, ζ', α^D^, α^3^, α^2^, α^1 ^[AC203513].

**Figure 3:**
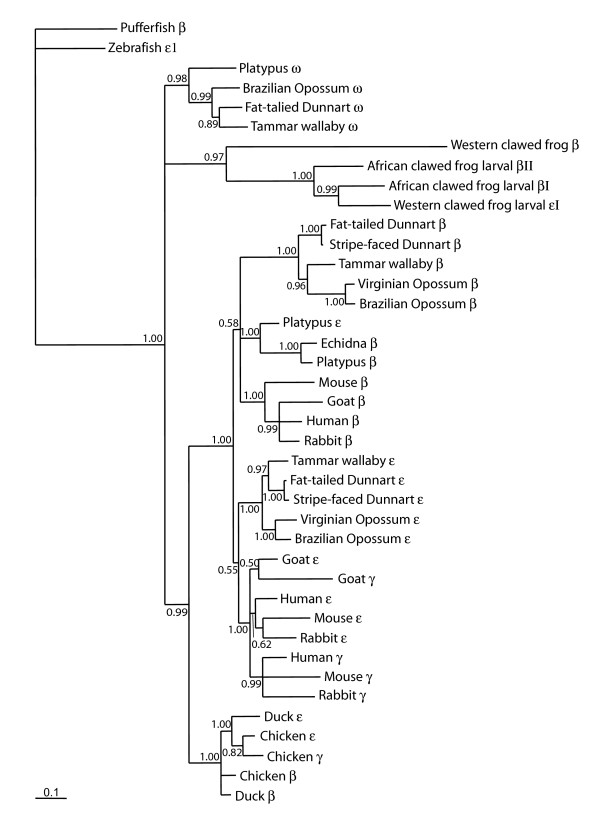
**Evolutionary relationships among vertebrate β-like globin genes using a 50% majority rule consensus phylogram from an analysis using Bayesian inference**. The tree was constructed using mixed models of evolution for each codon position (see methods) and estimated base frequencies in an unlinked analysis using MrBayes (v. 3.1.2). Numbers adjacent to branches refer to % posterior probabilities. GenBank accession numbers for sequences are: Fat-tailed Dunnart (*Sminthopsis crassicaudata*) β [Z69592], ε [Z48632], ω [AY014770]; Stripe-faced Dunnart (*S. macroura*) β, ε [AC148754]; Virginian Opossum (*Didelphis virginiana*) β [J03643], ε [J03642]; Brazilian Opossum (*Monodelphis domestica*) β [XM_001365299], ε [XM_001364448], ω [XM_001364828]; Tammar Wallaby (*Macropus eugenii*) β [AY450928], ε [AY450927], ω [AY014769]; African clawed frog (*Xenopus laevis*) larval β *I *[NM_001086273], larval βII [NM_001088028]; Western clawed frog (*X. tropicalis*) β [NM_203528], larval ε1 [NM_001016495]; Chicken (*Gallus gallus*) β (β^A^) [NM_205489], ε [NM_001004390], γ (β^A^) [NM_001031489]; Duck (*Cairina moschata*) β [J00926], ε [X15740]; Human (*Homo sapiens*) β [NM_000518], γ [BC130459], ε [NM_005330]; Mouse (*Mus musculus*) β (β1) [NM_008220], γ (β h0) [NW_001030869], ε (ε^y^) [M26897]; Goat (*Capra hirus*)*β *(β^A^) [DQ350619], ε (ε^I^) [X01912], γ [M15388]; Rabbit (*Oryctolagus cuniculus*) β, γ, ε [M18818]; Echidna (*Tachyglossus aculeatus) *β [L23800]; Pufferfish (*Fugu rubripes*) β [AY170464]; Zebrafish (*Danio rerio*) ε1 [NM_001103130]; Platypus β, ε [AC192436], ω [AC203513].

**Figure 4:**
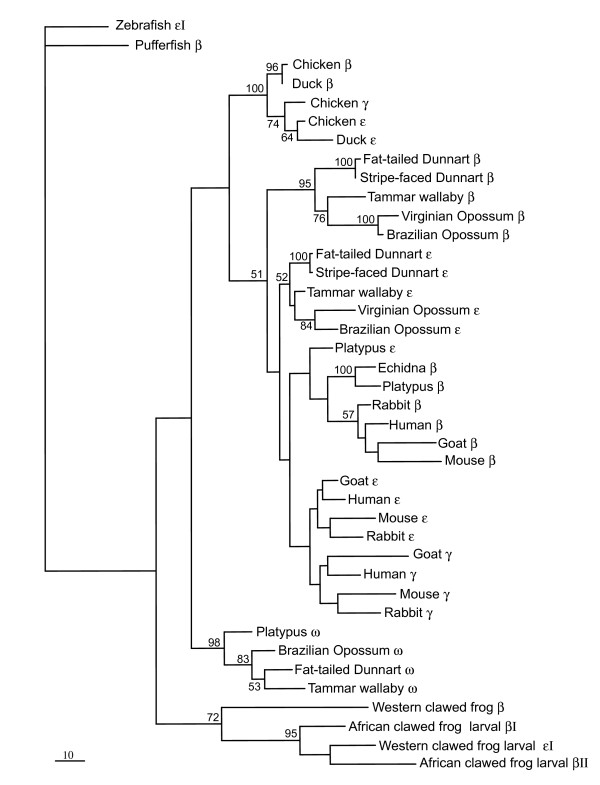
**Evolutionary relationships among vertebrate β-like globin genes analysed by maximum parsimony (MP) trees of length 926 (one of eight trees)**. Third position in codons were excluded in the MP analyses, which were conducted using a heuristic search in PAUP* v.4.0b10 [65]. The tree is rooted using pufferfish β-globin. Numbers adjacent to branches represent % bootstrap values (>50%) from MP heuristic analyses of 1000 pseudoreplicates. Accession numbers for sequences are given in the caption of Figure 3.

Genes in these sequenced BAC clones were predicted by programs GENSCAN [46] and GenomeScan [47]. Many genes were predicted, which were then used for BLAST searches of nucleotide (BlastN) and amino acid (BlastP) databases to help identify them (data not shown). Phylogenetic analyses were also conducted for the platypus α- and β-like globin genes to further verify the identity of each gene (see below and also Figures 2, 3 and 4 below). With only one exception (platypus ε-globin, see below), the identities of all of the genes inferred by BLAST analyses were supported by phylogenetic analyses with high posterior probabilities and bootstrap support values.

### Predictions and characterisation of genes in the platypus α-globin cluster

One BAC (Oa_Bb-2L7) contained two embryonic α-like globin genes, and a second BAC (Oa_Bb-131M24) contained six α-like globin genes and a β-like globin gene (see Additional file 1). These two BACs were found to overlap by 10,066 base pairs (bp), resulting in a contig of 330,126 bp that contained the entire platypus α-globin cluster and flanking genes.

**Figure 5:**
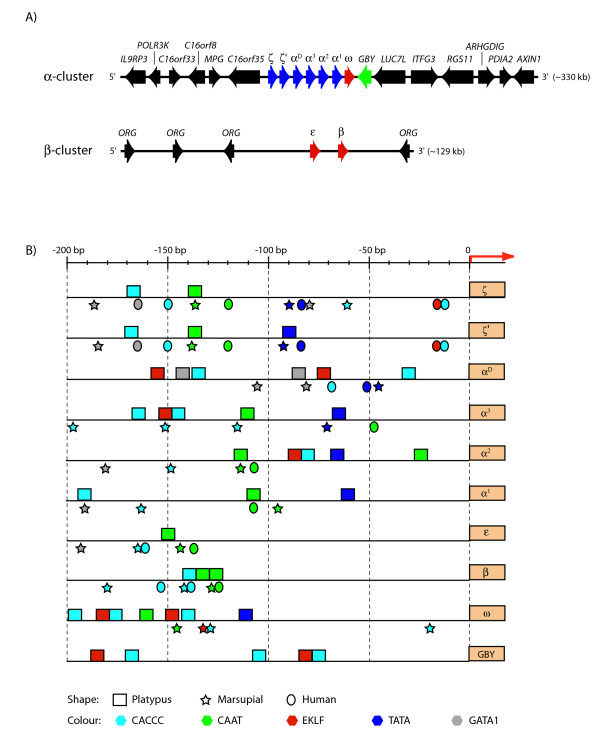
**Gene structure of the platypus α- and β-globin clusters and flanking loci, and comparisons of their promoter regions with other mammals**. (A) The platypus α-globin cluster contains six α-like globin genes (red), a β-like (ω) globin gene (blue) and a distantly related globin gene, *GBY *(green), which are flanked by *IL9RP3-POLR3K*-*C16orf33*-*C16orf8-MPG-C16orf35 *on the 5' end and *LUC7L-ITFG3-RGS11*-*ARHGDIG*-*PDIA2*-*AXIN1 *on the 3' end (black). The platypus β-globin cluster contains only two genes, ε and β (blue), which are flanked on both sides by *ORG *genes (black). (B) Relative positions of the putative transcription factor binding sites in the 200 bp promoter region located upstream of the predicted platypus, marsupial (*Didelphis virginiana *ζ and ψζ', and *Sminthopsis macroura *α^D^, ψα^3^, α^2^, α^1^, ω, ε and β) and human α- and β-like globin genes. For the platypus *GBY *no data was available from other species, including *Xenopus tropicalis*, for comparisons.

The 330,126 bp α-globin contig was found to contain six α-like globin genes, a β-like globin gene, and a gene that bore little similarity to α- and β-like globin genes but some similarity to cytoglobins (Figure 5A). These six α-like globin genes have a three-exon/two-intron structure and conserved donor/acceptor splice sites (GT/AG) typical of all vertebrate α-like globin genes. They are separated from each other by 2 to 6 kilobase pairs (kb). Full details of the exon/intron lengths, location of the putative poly-A addition site (AATAAA) and the lengths of the coding domains with the predicted encoded polypeptide for each predicted gene are given in Table 1. Figure 5B shows the predictions for some of the well-characterised protein-binding sites in the 5' promoter region (about 200 bp 5' to the cap site of each gene). These include CACCC [48], CAAT [49], TATA [50], GATA 1 [51], EKLF (Erythroid Krüppel-like Factor; [52]) and have been experimentally shown to control the stage- and tissue-specific expression of α- and β-like globin genes in other mammals [50,53-55].

**Table 1:** Gene-structure of the predicted platypus α- and β-like globin genes and *GBY*

**Length/** **Genes**	**Exon 1 ** **(bp)**	**Intron 1 ** **(bp)**	**Exon 2 ** **(bp)**	**Intron 2 ** **(bp)**	**Exon 3 ** **(bp)**	**Position of ** **Poly-A**	**CDS ** **(bp)**	**Poly- ** **peptide ** **(aa)**
**ζ**	95	337	205	114	129	+119	429	142
**ζ^1^**	95	336	205	102	129	+133	429	142
**α^D^**	92	1450	205	1610	129	+77	426	141
**α^1^**/**α**^**3**^	92	405	205	151	129	+94	426	141
**α^2^**	95	720	205	155	129	+115	429	142
**ω**	92	256	223	111	129	+69	444	147
***GBY***	98	3364	223	3053	144	+141	465	154
**ε**	92	143	223	474	129	+96	444	147
**β**	92	153	223	438	129	+71	444	147

For each predicted gene, the length of the exons and introns, position of poly-A addition site (AATAAA) from the stop codon, and the length of their putative coding domain (CDS) and encoded polypeptide are shown. All genes contained consensus splice sites (GT/AG) in both introns.

Two genes at the 5' end of the α-globin cluster were both identified as ζ-like (referred to here as ζ and ζ') and predicted to encode polypeptides of 142 amino acids (aa), which are typical of known functional mammalian α-like globin genes. The amino acid sequence alignment of ζ and ζ' shows 95% identity. In the promoter region of both genes, CACCC and CAAT consensus boxes are conserved at similar positions, and in comparable order to that of human ζ and ζ' (Figure 5B).

Adjoining the two ζ-like globin genes, four other α-like globin genes were identified. One was an orthologue of bird and reptilian α^D^, and the other three were orthologues of adult α genes (here called α^3^, α^2 ^and α^1^). The long and uninterrupted open reading frame (ORF) of α^D ^strongly suggests that it encodes a functional polypeptide of 141 aa, typical of known functional α^D ^globin genes. The platypus α^D ^globin gene contains introns of 1450 bp (intron 1) and 1610 bp (intron 2) that are very large compared with those of other α-like globins, which are usually less than 1000 bp.

Analyses of the platypus adult α-like globin genes reveal three adult (α^3^, α^2 ^and α^1^) globin genes in the α-globin cluster. The sequence of α^3 ^(the most 5' gene, adjacent to α^D^) was found to be almost identical to α^1 ^(the most 3' gene) in their exon and intron regions, as well as in flanking regions of about 130 bp on both sides. The coding region was 100% identical, and just two sites in intron 1 were found to be different between the two genes. In order to confirm that identification of these two identical genes was not due to an error in the assembly of the original sequence data, the boundaries of the region containing the homology between α^1 ^and α^3 ^was further analysed by a BLAST search of the platypus whole-genome shotgun (WGS) database (data not shown). Two contigs were identified with homology to α^1 ^and α^3^; these had identical sequences on one side of the boundary but different sequences on the other, confirming the presence of two separate genes. Further confirmation was obtained by performing a Southern blot on the α-globin-containing BACs, digested with an enzyme (*EcoRV*) that does not cut within the α^1^, α^2^and α^3 ^(data not shown). Probing with α^1^/α^3 ^revealed two bright bands, corresponding to α^1 ^and α^3^, and one fainter band between them, corresponding to α^2^. Probing with α^2 ^produced the same three bands, but in this case the middle one was brighter, corresponding to α^2^, and the outer bands were fainter, corresponding to α^1 ^and α^3^. These analyses confirmed the existence of separate genes α^1 ^and α^3 ^in the platypus α-globin cluster. The α^2 ^gene, located between α^1 ^and α^3^, was distinct from both genes in the coding sequence (with 83% homology), in intron lengths (intron 1: 405 bp in α^1^/α^3 ^and 720 bp in α^2^; intron 2: 151 bp in α^1^/α^3 ^and 155 bp in α^2^) and in the promoter region (Figure 5B).

The amino acid sequence encoded by α^1 ^and α^3 ^was identical to the platypus adult α-chain previously identified by Whittaker and Thompson [41], implying that at least one of these genes is expressed in the adult platypus. The coding domain of α^1 ^and α^3 ^is shorter (426 bp) than that of α^2 ^(429 bp), because it lacks the first three nucleotides of exon 1. The ORF of α^2 ^gives a strong indication that it is translated into a functional polypeptide of 142 aa, typical of known functional mammalian α-like globin genes.

On the 3' side of the six α-like globin genes, a β-like globin gene was predicted, which was identified as the orthologue of the marsupial ω-globin gene. This platypus ω-globin gene has a typical three-exon/two-intron structure, conserved donor/acceptor splice sites, and encodes a polypeptide of 146 aa, typical of all vertebrate β-like globin genes (Table 1). The promoter region located 5' of the ω-globin initiation codon contains conserved sites for CAAT-EKLF-CACCC in an order identical to that of marsupial ω-globin gene.

Unexpectedly, GenomeScan predicted a gene based on the protein similarities with the α- and β-polypeptide chains, approximately 1.5 kb 3' of the ω-globin gene. Like other α- and β-globins, this gene also has a three-exon/two-intron structure and conserved donor/acceptor splice sites (Table 1). The lengths of its exons 1, 2, and 3 are 98, 223 and 144 bp, respectively, compared with 92, 223 and 129 bp in other β-like globin genes. However, it has much larger introns of 3364 bp (intron 1) and 3053 bp (intron 2). The long and uninterrupted ORF of this gene can be translated into a polypeptide of 154 aa, which is atypical of any known α- or β-like globin genes. A BLAST search of the amino acid sequence of this gene obtained the best hit with Globin Y (*gby*) of the amphibian *X. laevis *(identity score of 39%), and weaker identity scores with Cytoglobins (*cygb*) of other species, such as the fish *Danio rerio *(27%), *X. tropicalis *(26%), chicken (28%) and human (25%) at the protein level. We designated this gene '*GBY*' based on similarities with *X. laevis gby*, and its similar position adjoining the globin cluster [2]. The predicted polypeptide of platypus *GBY *(154 aa) was shorter than *X. laevis gby *(156 aa), and quite different from *X. laevis cygb *(179 aa), *D. rerio cygb1 *(174 aa) and *cygb2 *(179 aa), and human *CYGB *(190 aa). Using the Expressed Sequence Tag (EST) database, a BLAST search of the platypus *GBY *also obtained an identity score of 38% with *X. tropicalis gby *that was expressed in both tadpoles and adults, but produced no significant matches with any other mammalian genes. The present work was the first opportunity to analyse the promoter region of any *GBY *gene (Figure 5B).

### Predictions and characterisation of genes in the platypus β-globin cluster

In the platypus, only two β-like globin genes were predicted within the 129,521 bp BAC clone (Oa_Bb-484F22) by GENSCAN and GenomeScan (see Additional file 1). When the predicted amino acid sequences were subjected to BLAST search, the 5' gene had best hits with mammalian embryonic ε-globin genes. Although the phylogenetic analyses using Bayesian inference (BI; see below) indicated that this gene was more closely related to the platypus and echidna adult β-globin genes than to therian ε-globin genes, the position of this gene on the 5' end of the β-globin cluster and expression data (see below) supports its orthology with mammalian embryonic ε-globin genes, and is henceforth referred to as ε. The 3' gene encoded a protein identical to the previously identified platypus adult β-chain [42], and is henceforth referred to as β.

Both genes encode polypeptides of 146 aa, typical of known functional mammalian β-like globin genes. The promoter region of the platypus β has conserved sites of CACCC and CAAT in all three extant of mammals. However, the promoter region of the platypus ε appears to be quite different from other mammalian ε-globin genes and even from the platypus β (Figure 5B). The promoter of platypus ε contains only one predicted motif (CAAT), whereas the promoters of other mammalian ε, β and the platypus β contain many predicted motifs.

### Expression studies of the platypus α- and β-like globin genes

Transcription studies were performed to gain insight into the expression and function of all of the predicted platypus globin genes. Adult liver, kidney, spleen, testis, lung and brain were obtained for this project: no embryonic samples were available (or are ever likely to be available) for this vulnerable and iconic species. Observation of the expression of any of the predicted genes would constitute a good indication that the gene is transcriptionally active and functional.

**Figure 6:**
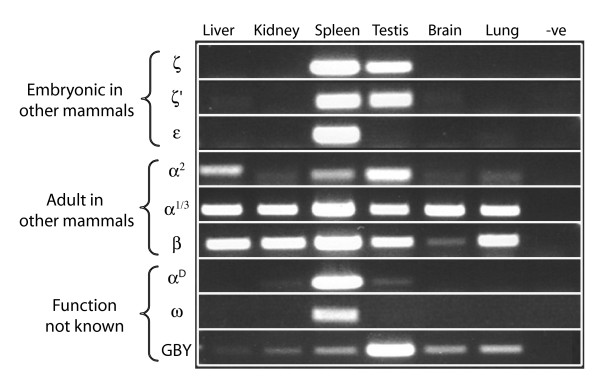
**Expression of all predicted α- and β-like globin genes including *GBY *in an adult platypus**. For each of the platypus predicted genes, expression was investigated by reverse transcriptase polymerase chain reaction in adult liver, kidney, spleen, testis, brain and lung. Primers for each gene were designed between two exons so that it would result in a product distinguishable from genomic contamination of cDNA. The negative control (last lane) contained no cDNA. All genes were expressed in one or more tissues, indicating that they are transcriptionally active and might be functional.

Reverse-transcriptase polymerase chain reaction (RT-PCR) of all predicted platypus genes showed that they are all expressed in at least some of these adult platypus tissues (Figure 6). Platypus genes α^1^/α^3^, α^2 ^and β, whose orthologues are usually expressed in the bone marrow of an adult human, were expressed in almost all platypus tissues tested, suggesting a broader expression of these genes in the monotreme lineage. Surprisingly, the genes ζ, ζ' and ε, whose therian orthologues are expressed only at embryonic stages of development, were expressed in adult spleen and testis, but not in the other tissues of adult platypus. This suggests that persistent expression of these genes in some adult tissues was selected for in the platypus, perhaps in response to its aquatic lifestyle and the hypoxic conditions of a confined burrow. Also, the expression pattern of platypus ε is similar to embryonic α-like ζ and ζ' but different from that of adult globin genes (α^1^/α^3^, α^2 ^and β). The ω and α^D ^globin genes, whose functions are unknown, were also expressed mainly in the spleen. *GBY *was expressed in all adult platypus tissues, most strongly in testis.

### Phylogenetic analyses

Phylogenetic analyses of the α-like globin genes using BI and maximum parsimony (MP) produced several noteworthy results. The platypus adult α globin genes (α^1^/α^3 ^and α^2^) grouped closely together to the exclusion of eutherian and marsupial α- and θ-globin genes for all analyses, although posterior probability (69%) and bootstrap support (66%) for this arrangement were relatively weak (Figure 2). This finding suggests that the duplication leading to the marsupial and eutherian θ-globin lineage occurred after the divergence of the monotreme and therian lineages. This is consistent with the absence of a θ-globin gene from the region between platypus α^1^- and ω-globin, its expected location based on its position in marsupial α-globin clusters [12,56].

Both platypus ζ-globin genes grouped closely together and formed a sister group relationship with chicken π, supported by a high posterior probability of 97% (Figure 2). A sister group relationship was also found in MP trees for analyses of the entire platypus coding region (bootstrap support <50%), and when third positions in the codon were excluded, was supported by 73% bootstrap pseudoreplicates (data not shown). This differs from the expectation that platypus ζ-globin genes would group with other mammalian ζ-globin genes to the exclusion of chicken π, suggesting that other factors (for example, purifying selection) operated to maintain a similar sequence in birds and monotremes.

There is still considerable uncertainty in the phylogenetic position of the α^D^-globin clade. It has recently been proposed that the α^D ^globin lineage resulted from duplication of the embryonic α-globin lineage, with phylogenetic analyses supporting a sister lineage relationship of these lineages to the exclusion of the adult α-globin lineage [14]. However, this arrangement was not supported in BI analyses of the data set used here, and the position of the α^D ^lineage was different in the different analyses. Analyses using BI (Figure 2) supported the sister lineage relationship of the α^D ^and adult α-globin lineages (as proposed by Cooper et al. [12]), with 87% posterior probability support. In contrast, all MP analyses supported the sister lineage status of α^D ^and embryonic α-globin genes, indicating an uncertainty in the phylogenetic position of the α^D^-globin clade.

Phylogenetic analyses of the β-globin genes provided results similar to recently reported phylogenetic analyses [35,36], with one notable exception. The BI analyses of coding sequence data (Figure 3) provided strong support (99% posterior probability) for the sister relationship of bird and mammalian β-like globin genes, contradicting previously published phylogenies of mammalian β-globin genes showing a sister relationship of marsupial ω-globin and bird β-like globin genes [35,36]. MP analyses (Figure 4), excluding third position in the codon, gave a similar tree arrangement, albeit with very low bootstrap support (<50%). In marked contrast to the BI analyses of DNA sequence data, BI protein analyses (data not shown) supported the sister relationship of bird β-like globin and mammal ω-globin lineages with a high posterior probability (99%).

Lastly, phylogenetic analyses using BI indicated that the platypus ε gene was more closely related to the platypus and echidna adult β-globin genes than to therian ε-globin genes, suggesting it may not be orthologous to marsupial and eutherian ε-globin (Figure 3). BI analyses of β-globin protein data and MP analyses of the coding sequence data, with third codon positions excluded, grouped the gene as an ancestral lineage to eutherian and monotreme adult β-globin genes (see Figure 4). This ancestral position suggests that the lineage evolved following duplication of an ancestral β-globin gene prior to the divergence of monotremes and therians.

### Location of the α- and β-globin clusters in the platypus

**Figure 7:**
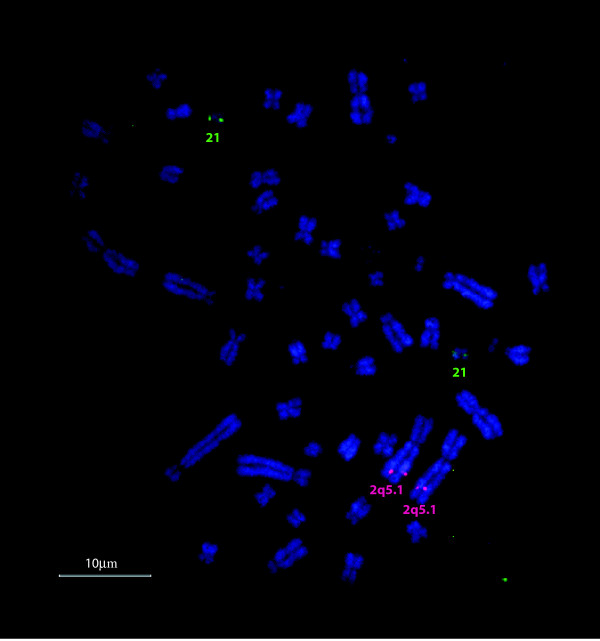
**Chromosomal location of the platypus α- and β-globin clusters**. Two-colour fluorescence in situ hybridisation showing the location of the α-globin cluster on chromosome 21 (green) and the β-globin cluster on chromosome 2q5.1 (red). The chromosomes are counterstained with DAPI (blue).

The location of the verified BAC clones containing the α- (Oa_Bb-2L7) and β-globin (Oa_Bb-484F22) clusters in the platypus was determined by fluorescence *in situ *hybridisation (FISH) (Figure 7). The β-globin cluster localised to one of the largest autosomes, giving unambiguous signals on the long arm of chromosome 2 (2q5.1). The α-globin cluster localised to the smallest autosome, 21, whose two arms are not distinguishable by size or DAPI banding pattern [21]. This is the first gene that has been localised on the platypus chromosome 21.

### Loci flanking the α- and β-globin clusters in the platypus and other vertebrates

To explore the genome context of the α- and β-globin clusters in the platypus and other vertebrates, the platypus BAC sequences and the genomes of other sequenced species were searched for loci residing beside the α- and β-globin clusters.

As well as globin genes, GENSCAN predicted within the platypus α-globin 330,126 bp contig many genes that flank the platypus α-globin cluster (Figure 5A), which were identified by BLAST analyses. These include *IL9RP3-POLR3K*-*C16orf33*-*C16orf8-MPG-C16orf35 *upstream (5') of the α-globin cluster, and, *LUC7L-ITFG3-RGS11*-*ARHGDIG*-*PDIA2*-*AXIN1 *downstream (3') of the α-globin cluster (Figure 5A).

**Figure 8:**
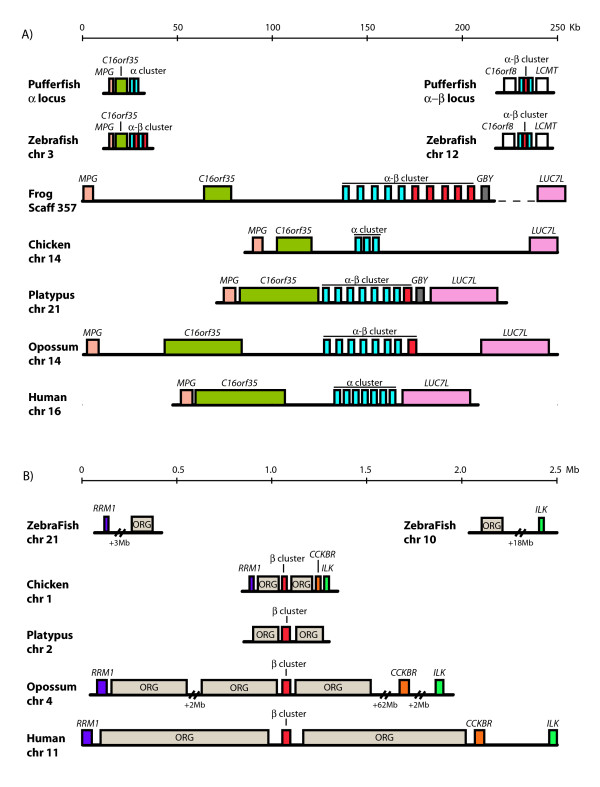
**Loci flanking vertebrate α-globin (A) and β-globin (B) clusters**. The relative locations of flanking loci (A) *MPG*, *C16orf35*, *LUC7L *and *GBY *and (B) *RRM1*, *CCKBR*, *ILK *and *ORG *genes were searched for beside the α-β globin cluster in zebrafish (*Danio rerio*) and frog (*Xenopus tropicalis*), and beside the separate α-globin and β-globin clusters in chicken (*Gallus gallus*), opossum (*Monodelphis domestica*) and human (*Homo sapiens) *from Ensembl [57]. The pufferfish (*Fugu rubripes*) flanking loci shown here were adapted from Gillemans et al. [8]. For the platypus, the α-globin flanking loci were characterised in this study, and *ORG *genes surrounding the platypus β-globin cluster were discovered: however, the BAC clone (484F22) was too small to cover the region containing the loci *RRM1*, *CCKBR *and *ILK*. In *X. tropicalis LUC7L *was found on another scaffold (466 from Ensembl) but sequence analyses by Fuchs et al. [2] suggested that *LUC7L *resides 3' to the frog α-β-*GBY *cluster. The flanking loci as well as the α- and β-globin clusters are differentiated by colour.

To compare the α-globin flanking loci of the platypus and other vertebrates, the genes closest to the α-globin cluster, *MPG*, *C16orf35 *and *LUC7L *were searched for in the human, opossum (*Monodelphis domestica*), chicken, frog (*X. tropicalis*) and zebrafish (*D. rerio*) genomes that were accessible from Ensembl [57]. Figure 8A shows that the locations of *MPG*, *C16orf35 *and *LUC7L *are conserved adjacent to the α-globin cluster of birds and mammals, and in the same position adjacent to the α-β cluster of amphibians, and all but *LUC7L *were also present in fish. These results are consistent with the previous analyses of Flint et al. [58] and Hughes et al. [59]. Thus the flanking loci analyses reveal that the genome context of the platypus α-globin cluster is the same as the α-globin clusters in therian mammals and birds, and this is the same as for the α-β cluster of fish and frogs.

GENSCAN also predicted numerous genes other than globin genes in the platypus β-globin BAC (484F22). These were identified by a BLAST search as members of the olfactory receptor gene (*ORG*) family that are responsible for odour detection. Three conserved *ORG *members were identified at the 5' end of the platypus β-globin cluster and one conserved *ORG *member at the 3' end (Figure 5A).

To compare β-globin flanking loci, *ORG *genes, as well as other genes that are closest to the β-globin cluster in other species, *RRM1*, *CCKBR *and *ILK *were searched for in the human, opossum, chicken and zebrafish genomes that were accessible from Ensembl [57]. Data from frog (*X. tropicalis*) was not useful since all of these loci lie on different contigs or scaffolds due to assembly problems. The locations of multiple *ORG *genes, *RRM1*, *CCKBR *and *ILK *were found to be conserved adjacent to β-globin cluster of birds and mammals [60,61], but not for the α-β cluster of fish and frogs, nor beside the second α-β cluster of zebrafish and pufferfish (Figure 8B). Thus the genome context of the platypus β-globin cluster is the same as in therian mammals and birds, but this is different from the α-β cluster of fish and frogs.

## Discussion

The phylogenetic position of monotremes makes comparisons with platypus of special value for exploring the organization, function and evolution of mammalian genes and genomes. The availability of platypus genome sequence data now makes many such studies possible, and have been used here to characterise the platypus α- and β-globin gene clusters and explore their evolutionary history.

### The platypus α-globin gene cluster

The platypus α-globin cluster contains at least eight genes within more than 40 kb, including six α-like globin genes (including the identical α^1 ^and α^3^), one β-like globin gene (ω-globin) and a gene belonging to another member of the globin super-family (*GBY*) arranged in the order 5'-ζ-ζ'-α^D^-α^3^-α^2^-α^1^-ω-*GBY*-3' (Figure 5A). The cluster maps to chromosome 21, the smallest autosome in platypus. All eight genes are likely to be functional since their expression was detected in tissues of an adult platypus.

**Figure 9:**
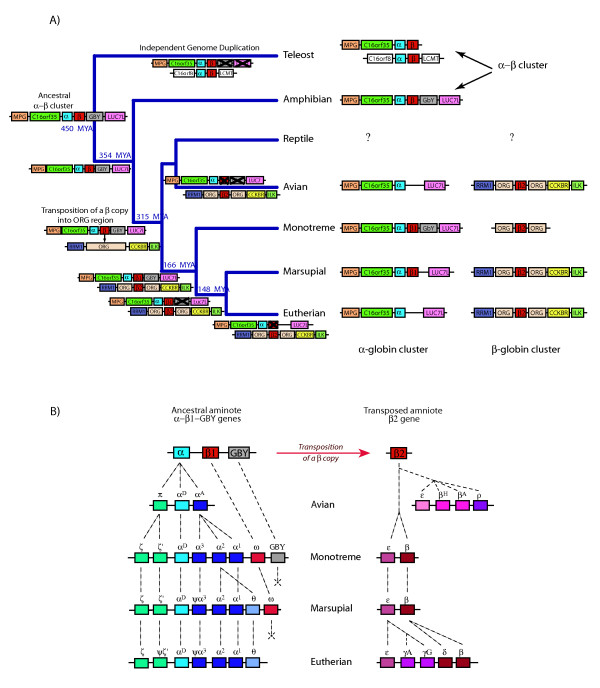
**Proposed model for the evolution of the α- and β-globin clusters in vertebrate lineages**. (A) A region containing *MPG-C16orf35*-α-β-*GBY*-*LUC7L *represented the ancient α-β globin cluster of jawed vertebrates (>450 MYA), which is seen in the amphibian lineage. This region further duplicated and underwent some gene silencing in teleost fish. In an amniote ancestor of reptiles, birds and mammals (>315 MYA), a copy of an ancestral β-globin gene from this region was inserted into a different chromosome within a region replete with multiple copies of *ORG *genes. The original amniote β-globin gene survives as the ω-globin gene (β1) in the α-globin cluster of marsupials and monotremes, whereas the transposed β-globin gene (β2) duplicated several times to form different clusters in the different lineages. (B) Tandem duplications of the ancestral amniote α-globin gene produced a three-gene (π-α^D^-α^A^) cluster in the avian lineage. In the mammalian lineage, further duplications gave rise to a six-gene (ζ-ζ'-α^D^-α^3^-α^2^-α^1^) cluster with ongoing gene conversion events homogenising the embryonic and adult genes. In monotremes, the ancestral ω (β1) and *GBY *are retained. After the divergence of monotreme and therian mammals, there was an additional duplication of α^2 ^to form θ, giving rise to the seven-gene cluster (ζ-ζ'-α^D^-α^3^-α^2^-α^1^-θ) in marsupials and eutherians. Marsupials also retain the ancestral ω but may have lost *GBY *gene; eutherians retain no identifiable remnant of either gene. Furthermore, the ancestral transposed β2-globin gene duplicated independently in birds and mammals. Before the mammalian radiation, we propose that the ancestral β2 gene duplicated to form a two-gene β-globin cluster (ε-β) as seen in monotremes and marsupials, except that ongoing gene conversion events homogenised platypus ε to group with monotreme β genes. After the divergence of marsupial and eutherian mammals, there were further tandem duplications of these two genes to produce complex β-globin cluster (ε-γ-η-δ-β) in eutherians.

The platypus α-globin cluster is almost identical to the arrangement of α-like globin genes in the ancestral therian cluster reported by Cooper et al. [12]. The one exception is the absence of a θ-globin gene from the platypus cluster. Phylogenetic analyses support the basal position of the monotreme adult α-globin lineage relative to marsupial and eutherian α- and θ-globin lineages, implying that the duplication of an adult α-globin to produce θ-globin occurred in the therian lineage after its divergence from the monotreme lineage (Figure 9B). However, although the numbers and arrangements of genes is so similar in platypus and therians, the presence of three adult α-globin genes and two embryonic ζ-globin genes in their common ancestor was not supported by phylogenetic analyses, which showed independent groupings of the three adult and embryonic genes within each separate mammalian lineage (Figure 2 and see Cooper et al. [12]). This result can be interpreted literally as resulting from independent duplications in each mammalian lineage to produce three adult and two embryonic genes in each. However, this seems unlikely to explain the convergence in gene number of the α-globin cluster in these distantly related mammalian lineages. We suggest that a more parsimonious explanation is that the common ancestor of monotremes and therians contained three adult α-globin genes and two ζ-globin genes, which were homogenised by ongoing gene conversion events, leading to the gene tree that does not match the duplication history of the individual genes. The close similarity of the platypus α^3 ^and α^1 ^loci suggests a very recent gene conversion event that homogenised their sequences. Therefore, we propose that the platypus α-globin cluster of eight genes (ζ-ζ'-α^D^-α^3^-α^2^-α^1^-ω-*GBY*) represents the ancestral mammalian α-globin cluster arrangement (Figure 9B), in which all genes were transcriptionally active.

Importantly, the platypus α-globin cluster contains a copy of the β-like ω-globin gene, also found in the marsupial α-globin cluster, but absent in humans, supporting the hypothesis that ω-globin was present in the common ancestor of all mammals. Phylogenetic analyses also confirm the ancient ancestry of the ω-globin gene, as concluded by Wheeler et al. [35,36]. Among adult platypus tissues this gene was expressed only in the spleen. In marsupials, expression of the ω-globin gene was detected just prior to birth and during early pouch young development [37], although the site of expression was not studied, and there was no evidence of adult expression in blood cells.

### Discovery of a mammalian GBY globin gene adjoining the α-globin cluster

We discovered a globin gene *GBY *in the platypus that is adjacent (3') to ω in the α-globin cluster. It has a typical three-exon/two-intron structure like other α/β-globin genes, contains an ORF encoding a polypeptide chain of 154 aa, and is expressed in almost all adult tissues, most strongly in testis. The amino acid sequence is unrelated to any of the other globin genes in the cluster, so it is unlikely to be derived by duplication of α- or ω-globin within the monotreme lineage. Rather, it shows sequence similarity to *gby *of *X. tropicalis *and *X. laevis*, a gene thought to be related to cytoglobins [2].

Little is known of the function of amphibian *gby*, or its relationship with other globins. Fuchs et al. [2] reported that amphibian *gby *encodes a *bona fide *globin of 156 aa, having all of the sequence features of a functional respiratory protein. *gby *was expressed in all adult tissues tested in *X. laevis*, most strongly in ovary, kidney and eye, and was present in 20 expressed sequence tag clones from different stages of *X. laevis *and *X. tropicalis *embryonic and adult development [2], suggesting that it is expressed in embryonic as well as adult stages. Phylogenetic analysis of all vertebrate globins [2] showed that the *gby *lineage diverged at the base of two separate clades, one comprising all vertebrate cytoglobins, myoglobins, agnathan globins and bird globin E, and the other comprising the haemoglobin α- and β-chains.

The position of platypus *GBY *adjacent to the α-globin cluster and flanked by *LUC7L *mirrors its position in *X. tropicalis *between the main α-β cluster and *LUC7L *[2]. Another common feature of both was strong expression in gonads (ovary in *X. laevis *[2] and testis in platypus), so *GBY *has sex-related expression in both lineages. Thus *GBY *is not specific to amphibians, as was thought, but was a component of the cluster in an ancient tetrapod, and has been lost, or has diverged beyond recognition, in birds and therian mammals.

### The platypus β-globin gene cluster

Characterisation of the platypus β-globin cluster revealed two β-like globin genes over about 13.2 kb that are arranged in the same order as marsupials, 5'-ε-β-3' (Figure 5A). This cluster is located on platypus chromosome 2q5.1. Both genes appear to be transcriptionally active and are likely to be functional.

At the time of revising this paper, an independent paper on monotreme β-like globin genes was published by Opazo et al. [62] in which they reported the presence of ω, ε^P ^and β^P ^in the platypus. Largely on the basis of phylogenetic analyses of flanking and coding sequence data, they proposed that platypus ε^P ^and β^P ^were not 1:1 orthologues of therian ε and β, respectively, and arose by independent duplication of an ancestral β-globin gene in the monotreme lineage, with a separate duplication event, just prior to the divergence of therians, producing the progenitors of ε and β of therians. This hypothesis was strongly supported by our BI phylogenetic (Figure 3) analyses, but not by MP analyses of coding sequence data, with third codon sites excluded (Figure 4), or BI analyses of protein sequence data (not shown). These contradictory analyses highlight the difficulty in resolving deep relationships among globin genes, particularly when the time periods between duplication and speciation events are relatively small, the phylogenetic signal at third codon positions is potentially saturated, and non-synonymous sites may be subjected to purifying or positive selection. Despite a very high posterior probability (100%) for the grouping of platypus ε with monotreme β, this value is a Bayesian probability and depends on the model adequately representing the evolution of the gene. Furthermore, although it was reported [62] that the 5' flanking sequences of platypus ε and β were similar, we found no evidence for similarity of the promoter signals of these two genes (Figure 5B).

We consider that a more parsimonious explanation is that the platypus ε is orthologous to the marsupial and eutherian embryonic β-like globin lineages (ε and γ), and arose by duplication of an ancestral β-globin gene prior to the mammalian radiation (166 MYA; Figure 9B). The sequence of platypus ε may have been homogenised by some gene conversion events, leading it to group with other monotreme adult β-like globin genes. In addition to the MP analyses reported above, this explanation is further supported by the conserved position of ε to the 5' side of the adult β-globin gene in the platypus cluster, which is similar to that found in other therian β-globin gene clusters [26]; see also [29]). Amino acid sequence analyses (BlastP) also provided additional support for the orthology of platypus ε to other mammalian ε-globin genes. Although we were unable to examine the expression of the genes in embryonic tissues, it was found that the expression profile of the platypus ε was similar to the embryonic α-like globins ζ and ζ' of the platypus, but not to the adult β-globin gene, supporting its potential role as an embryonic β-like globin gene.

### The ω-globin gene and the evolution of the β-globin cluster

The discovery of the marsupial ω-globin gene in the α-globin cluster [35,36] was critical in re-interpreting the relationships of the α- and β-globin clusters in amniotes (reptiles, birds and mammals) to favour the hypothesis that these clusters in birds and mammals are paralogous, having diverged independently from different ancestral copies of the vertebrate α-β-globin locus [63].

Our observation of an ω-globin gene in the α-globin cluster in the platypus, as well as in the marsupials, confirms that the ancestral mammal α-globin cluster contained a β-like globin gene that was lost in eutherians, as proposed by Wheeler et al. [35,36]. However, the position of monotreme and marsupial ω in the phylogeny (Figure 3) is more consistent with the original hypothesis [5] that mammal and bird β-globin are orthologous, having descended from the same β-globin progenitor in an amniote ancestor, and this is strongly supported by flanking sequence data (see below). Our data support the proposition that the ω *-*globin gene represents an ancient β-like globin gene lineage that is ancestral to a group containing both mammalian and bird β-globins with a high posterior probability (99%). This arrangement, however, was not supported by analyses of amino acid sequence data, indicating that there is uncertainty in the phylogenetic position of ω-globin relative to bird β-globins, or that convergent evolution of bird β-globin genes and ω-globin resulted in their similarity at the protein level. To further resolve the key question of whether bird and mammal β-globin gene clusters are orthologous we carried out comparative analyses of flanking loci of the α- and β-globin clusters.

### Genome context of vertebrate α- and β-globin clusters

We found that the platypus α-globin cluster is flanked by *MPG*, *C16orf35*, *GBY *and *LUC7L*, and that the same genes (except *GBY*) flank the α-globin cluster in mammals and birds [58,59]. The same genes flank the α-β cluster of frog, and even zebrafish and the α-cluster of pufferfish [8] (except *GBY *and *LUC7L*), implying that a very ancient region containing these genes (5'-*MPG*-*C16orf35*-α-β-*GBY*-*LUC7L*-3'), or perhaps an even larger region, was present in their common ancestor and has been conserved since the evolution of jawed vertebrates more than 450 MYA.

In contrast, the amniote β-globin clusters reside in a very different genome, sharing none of the flanking loci with the mammal and bird α-globin clusters, or the α-β cluster of frogs and fish. In platypus, as well as in therian mammals [60,61], the β-globin clusters are flanked by numerous *ORG *genes on both sides. In birds, also, the β-globin cluster is embedded in *ORG *genes [60]. Even the outside loci *RRM1*, *CCKBR *and *ILK *lie in the same orientation with respect to the bird and mammalian β-globin clusters [60], suggesting that the 5'-*RRM1-ORG-β *(cluster)*-ORG-CCKBR-ILK*-3' arrangement has been conserved since before the divergence of birds and mammals, more than 315 MYA. Therefore, the bird β-globin cluster is orthologous to the β-globin clusters of mammals.

## Conclusion

### New model for the evolution of α- and β-globin clusters in amniotes

This analysis of flanking loci, in addition to the phylogenetic analyses reported above, refutes the prevailing hypothesis that mammal and bird α- and β-globin clusters evolved from different (paralogous) copies of an ancestral α-β-globin region containing *MPG-C16orf35-α *(cluster)-β (cluster)*-GBY-LUC7L*. Rather, the context of β-globin clusters within olfactory receptor genes in birds as well as mammals suggests that a copy of a β-globin locus was moved into a region replete with *ORG *genes before the divergence of birds and mammals 315 MYA. The precise mechanism for this translocation is unknown, but is likely to be either by transposition of a tandem duplicate of an ancestral β-globin gene, or retrotransposition of an intron-containing primary transcript. Phylogenetic analyses suggest that this ancestral β-globin gene within the α-globin cluster is represented by the platypus and marsupial ω-globin gene. The transposed β-globin gene then independently duplicated several times within the avian and mammalian lineages to form the different clusters of differentially expressed β-globin genes. Full details of this new model are given in Figure 9A and 9B.

This hypothesis could be further tested by investigating the gene organization of the α- and β-globin clusters in reptiles such as lizards and snakes, which form a sister group to birds. Our hypothesis predicts that reptiles should possess a *MPG-C16orf35*-α (cluster)-β (cluster)*-GBY-LUC7L *cluster, and an unlinked *RRM1-ORG-β *(cluster)*-ORG-CCBKR-ILK *cluster like birds and mammals. The full genome sequence of the first reptilian species,*Anolis carolinensis*, will provide an opportunity to test this hypothesis.

## Methods

### Isolation and purification of probes to screen for platypus β-globins

At the start of this project there were no trace sequences available for any globin genes in the platypus trace archive. We therefore designed probes to screen the platypus male Oa_Bb BAC library (Clemson University Genomic Institute, USA). The platypus β-globin-specific primers OaBGF (5'-tggacccagaggttctttgac-3') and OaBGR (5'-tgcaattcactcagcttggag-3') were designed from the reference tammar β-globin sequence [GenBank: AY450928] using Primer3 [64]. Amplification by PCR was performed in a final volume of 25 μl, with 40 ng genomic DNA, 1× Buffer (Roche, Australia), 0.2 mM dNTPs, 0.05 U Taq (Roche, Australia) and 1 μM each of forward and reverse primers. PCR cycling conditions were: 94°C for 2 minutes, then 35 cycles of 94°C for 30 seconds, 50 to 60°C for 30 seconds, 72°C for 1 minute, followed by 72°C for 10 minutes. The PCR products were sub-cloned according to the TOPO TA cloning^® ^Kit Protocol (Invitrogen, Australia) and the resulting plasmids were purified according to the centrifugation protocol of Wizard^® ^Plus SV Minipreps DNA Purification System (Promega, Australia). The purified plasmids were confirmed to contain PCR products of a partial platypus β-globin gene (167 bp) by sequencing at the Australian Genome Research Facility (AGRF, Brisbane, Australia) using M13 forward (5'-gtaaaacgacggccag-3') and M13 reverse (5'-caggaaacagctatgac-3') primers. Once confirmed, they were used as probes to screen the platypus BAC library.

### Screening the platypus BAC library for β-like globin genes

The platypus BAC library filters were pre-hybridised at 65°C with Church Buffer (1 mM EDTA, 0.5 M phosphate buffer, 7% (w/v) SDS) including 1% BSA for 4 hours. The platypus β-globin probes (25 ng) were labelled with ^32^P-dATP using the Megaprime DNA labelling System (GE Healthcare, Australia) following the manufacturer's instructions. The probes were allowed to hybridise to the filters at stringent conditions (65°C with the above buffer) for 24 hours and then washed twice for 15 minutes each in 2 × SSC/0.1%SDS and 1 × SSC/0.1%SDS. Autoradiography was carried out for 14 days at -80°C with an intensifying cassette.

### Identification of platypus BAC clones containing α-like globin genes

Unlike β, BACs were not screened for α-like globin genes. Instead they were identified directly from the Encyclopaedia Of DNA Elements Project [65], in which the α-globin cluster is one of the targeted regions [12,66]. Two platypus BAC clones (Oa_Bb-2L7 and Oa_Bb-131M24), which were sequenced but not yet annotated, were identified by computational analysis (below) to contain parts of the α-globin cluster and a ω-globin gene.

### Isolation and purification of DNA from BAC clones

DNA from the identified BAC clones (including those that were screened) was extracted using Wizard^® ^Plus SV Minipreps DNA Purification System (Promega, Australia). The purified BAC clones were then subjected to Dot or Southern Blot to confirm the presence of α- or β-globin genes respectively.

### Confirmation of BACs containing globin genes

Dot blot methods were used to verify the presence of the α-like globin genes. In a plate containing Luria broth agar with chloramphenicol, a Hybond N^+ ^(GE Healthcare, Australia) filter was placed and multiple 1 μl of liquid culture BAC clones were spotted onto the filter. The plate was incubated at 37°C overnight and then the filter was soaked in Denaturation Solution (0.5 M NaOH and 1.5 M NaCl) for 5 minutes, followed by soaking twice in Neutralisation Solution (0.5 M Tris-Cl pH 7.4 and 1.5 M NaOH) for 5 minutes each. The filter was then rinsed in 2 × SSC, soaked in 0.4 M NaOH for 20 minutes and washed with 6 × SSC to remove all cellular debris. The filters were then screened with the platypus α-globin probes using the standard library screening procedure (above).

Southern blotting was used to verify the presence of the β-like globin genes. In a 40 μl reaction, 20 to 40 ng BAC DNA was digested with 10 U of restriction enzyme,*HIND III *(Roche, Australia). The reaction was incubated at 37°C for at least 4 hours and separated by electrophoresis on a 0.8% agarose gel overnight at 40 V. The DNA fragments were transferred onto a Hybond N^+ ^(GE Healthcare, Australia) nylon filter overnight by capillary action following the manufacturer's instructions, and cross-linked in 0.4 M NaOH for 20 minutes. These filters were then screened with the platypus β-globin probes using the standard library screening procedure (above).

### Fluorescence *in situ* hybridisation (FISH)

Male platypus metaphase spreads were prepared and *in situ *mapping was performed using two-colour FISH as described previously by McMillan et al. [21]. The verified BACs containing the α-like globin genes (ζ and ζ': Oa_Bb-2L7) and β-like globin genes (ε and β: Oa_Bb-484F22) were labelled with different fluorochromes and then hybridised to the chromosomes. The signals were detected by fluorescent microscopy, where at least twenty metaphase images were captured and analysed.

### Sequence data of the platypus BAC clones containing the α- and β-globin clusters

Information about the platypus BAC clones containing the α-like globin genes along with the ω-globin gene was obtained directly from the ENCODE Project [66]. Their sequence information was obtained from GenBank; accession numbers: AC195438 (Oa_Bb-2L7) and AC203513 (Oa_Bb-131M24).

The BAC clone containing the β-like globin genes that were found from the library screening procedure were sequenced at the Washington University Genome Sequencing Centre (St Louis, USA). The sequence information for this BAC clone was obtained from GenBank: AC192436 (Oa_Bb-484F22).

### Computational characterisation of the α- and β-globin clusters in the platypus

Using sequence information of AC195438, AC203513 and AC192436, genes were predicted by GENSCAN [46] and GenomeScan [47] using default settings. All predicted gene sequences were then subjected to a BLAST search of the translated nucleotide acid (BlastX) and protein (BlastP) databases to confirm their identities.

### Promoter analyses

Transcription factor binding motifs were predicted in the 200 bp promoter region located 5' to the predicted platypus α- and β-like genes and *GBY *by rVista 2.0 [67] using user-defined consensus sequences for 'CACCC', 'CAAT', 'TATA', GATA1 ('WGATAR' [51]) and EKLF ('NGNGTGGGN' [51]). The same criteria were used to predict the same motifs in marsupials (*Didelphis virginiana *[ζ and ψζ': AC139599] and *Sminthopsis macroura *[α^D^, ψα^3^, α^2^, α^1^, ω: AC146781; and ε, β: AC148754]) and in humancs [ζ, ψζ', α^D^, ψα^3^, α^2^, α^1^: NG_000006; and ε, β: NG_000007] for consistency in comparison.

### Confirmation of α1 and α3 by BLAST search and Southern blot

To confirm that the presence of two almost identical genes (α^1 ^and α^3^) was real rather than an assembly error, the boundaries (~300 bp) of the homologous regions were investigated by a BLAST search against the platypus WGS database. The raw sequences of best hits were extracted from NCBI GenBank, cleaned and aligned in Sequencher v4.8 (Gene Codes Corporation, Michigan) using default settings.

Southern blotting was also used to verify the presence of α^1 ^and α^3 ^genes. In a 30 μl reaction, 100 μg BAC DNA (Oa_Bb: 131M24, 130N2, 150K14 and 223I12) was digested with 10 U of restriction enzyme,*EcoRV *(Roche, Australia). The reaction was incubated at 37°C for at least 4 hours and separated by electrophoresis on a 0.8% agarose gel overnight at 40 V. The DNA fragments were transferred onto a Hybond N^+ ^(GE Healthcare, Australia) nylon filter overnight by capillary action following the manufacturer's instructions, and cross-linked in 0.4 M NaOH for 20 minutes. These filters were then screened with the platypus α^1^/α^3 ^(test) and α^2 ^(control) probes using the standard library screening procedure (above).

### RT-PCR analyses

To remove DNA contamination, RNAs derived from adult male platypus liver, kidney, spleen, testis, brain and lungs were DNase treated using a DNA-*free*™ kit according to the manufacturer's instructions (Applied Biosystems, Australia). Treated RNAs were then reverse transcribed using Superscript III (Invitrogen, Australia) following the manufacturer's instructions. Primers were designed against predicted α- and β- like and *GBY *globin gene sequences using Primer3 [64]. In each case, the region amplified spanned an intron so that the origin of the template (gDNA or cDNA) was immediately obvious. Primer sequences and the expected sizes of amplified cDNA and gDNA bands are shown in Table 2. PCR reactions and cycling conditions were the same as for screening for the platypus β-globin genes, above. The positive bands were directly sequenced by AGRF (Brisbane, Australia) to confirm their identities. The blood contamination in the tested samples had minimal effect on the observed expression pattern, as some tissues (for example, lung and liver) showed no amplification despite containing large quantities of blood.

**Table 2:** PCR primers used for amplification of the α- and β-like globin genes including *GBY *from the platypus gDNA and cDNA

**Gene**	**Forward Primer**	**Reverse Primer**	**gDNA** **(bp)**	**cDNA ** **(bp)**
**ζ**	GGCCGACAAGACCGCAGTCATCTCCC	CCCGATGGCGCTGATGACT	527	190
**ζ^1^**	TGACCAAAGGCGACAAGACCT	CCCCGATGGCACCGATGACC	534	198
**α^D^**	GAGGCTGTGAAGAACCTGGA	GGTGTACTCCCCTTGCAGAT	1793	153
**α^2^**	TGGCCCACCTCGATGACCTGG	GGGAAGGTGTCTGGCCACC	289	134
**α^1^**/**α**^**3**^	GCAAGGCCGCCGGTCACGGC	CGCTGTCCATGTCATCGAAGTGCC	597	192
**ω**	ATTGTGTCCATCTGGGGAAA	GCTTGGCAAAGTTGCTCTTC	488	232
***GBY***	CTGGAAACAGGTGTGCAAGA	CTATCTCCGGGGTGTAGCAG	3202	149
**ε**	ATCTGAGCGCTGAGGAGAAG	GACAGGTTGCCGAAGGAGTCA	285	142
**β**	CTGTGGGGGAAAGTGAACAT	GGTCAGCACCTTAGCACCAT	321	168

### Phylogenetic analyses

Phylogenetic analyses were employed to verify the identities of the platypus globin genes and study the evolutionary relationships of the different members of the α- and β-globin gene families. This study was restricted to the coding domains of the α- and β-globin members and the accession numbers of the sequences used are given in the legends of Figures 2 and 3. Phylogenetic analyses were conducted using MP in PAUP* v.4.0b10 [68], and a BI approach using MrBayes v.3.1.2 [69]. Concordance of trees from each of the different methods, bootstrap proportions and posterior probability estimates were used to examine the robustness of nodes.

MP analyses were conducted for the entire coding sequence matrix and after excluding third codon positions using a heuristic search option and default options (TBR branch swapping), with the exception of using random stepwise addition repeated 100 times. Character state optimisation for MP trees used the DELTRAN option. MP bootstrap analyses [70] were carried out using 1000 bootstrap pseudoreplicates, employing a heuristic search option with random stepwise addition.

The program MODELTEST [71] and the Akaike information criterion (AIC) were used to assess the most appropriate model for BI analyses. The MODELTEST analyses were facilitated using the program MrMTgui v1.0 [72]. The MODELTEST analysis was carried out on separate codon positions for α- and β-globin data sets. For α-globin sequences, a general time reversible (GTR) model [73], with a proportion of invariant sites (I) and unequal rates among sites [74], modelled with a gamma distribution (G) was found to be the most appropriate model to use for first and second codon positions, and a GTR+G model was appropriate for third codon positions under the AIC. For β-globin sequences a GTR+I+G model was considered appropriate for first positions, and a GTR+G model was found to be appropriate for second and third codon positions. The MrBayes analysis was carried out applying these different models to each codon position using an unlinked analysis, with default uninformative priors. Four chains were run simultaneously for 2 million generations in two independent runs, sampling trees every 100 generations. The program TRACER (version 1.3; [75]) was used to assess tree and parameter convergence. For both the α-globin and β-globin analyses all effective sample sizes for all parameters were >1297, indicating a sufficient sample of the parameter space had been taken. A burn-in of 2000 trees (equivalent to 200,000 generations) was chosen for each independent run of MrBayes, with a >50% posterior probability consensus tree constructed from the remaining 36,002 trees (18,001 trees each run).

A BI analysis using MrBayes (version 3.1.2) was also carried out using protein sequence data from β-globin genes. A mixed protein model was used, allowing the optimum model of protein evolution to be assessed from a selection of nine fixed-rate models. The optimum model was found to be the Dayhoff model with a posterior probability of 1.0. The analyses were conducted using two million generations in two independent runs, sampling trees every 100 generations. A burn-in of 2,000 trees was used for each run with a 50% consensus tree constructed from the remaining 36,002 trees.

## Authors' contributions

VSP designed and performed most of the experiments and analysed the data. VSP also drafted the main manuscript. SJBC conducted phylogenetic analyses and contributed to the writing of the manuscript. JED helped in designing the experiments and trouble-shooting experiments. BF, TG, WCW and RKW were involved in sequencing the platypus BAC clone (Oa_Bb-484F22). JAMG conceived and supervised the research and contributed to the writing of the manuscript. All authors read and approved the final manuscript.

## Competing Interests

The authors declare that they have no competing interests.

## Supplementary Material

Additional file 1 **Annotation of the platypus α- and β-like and *GBY *globin genes**. This table shows the predicted positions of six α-like, ω and *GBY *globin genes in the platypus BAC clone Oa_Bb-131M24 [GenBank: AC203513], two α-like globin genes in BAC clone Oa_Bb-2L7 [GenBank: AC195438], and two β-like globin genes in BAC clone Oa_Bb-484F22 [GenBank: AC192436 reverse direction]. Click here for file
