# Supplementary material for: Platypus globin genes and flanking loci suggest a new insertional model for beta-globin evolution in birds and mammals
Source: BMC Biol. 2008 Jul 25;6:34. doi: 10.1186/1741-7007-6-34 (PMC2529266; doi:10.1186/1741-7007-6-34)
Supplement: Additional file 1 — Annotation of the platypus α- and β-like and GBY globin genes. This table shows the predicted positions of six α-like, ω and GBY globin genes in the platypus BAC clone Oa_Bb-131M24 [GenBank: AC203513], two α-like globin genes in BAC clone Oa_Bb-2L7 [GenBank: AC195438], and two β-like globin genes in BAC clone Oa_Bb-484F22 [GenBank: AC192436 reverse direction]. [file 1741-7007-6-34-S1.doc]

Additional File

**Additional File 1: Annotation of the platypus α- and β- like and *GBY* globin genes**

The table shows the predicted positions of six α-like, ω and *GBY* globin genes in the platypus BAC clone Oa_Bb-131M24 [GenBank: AC203513], two α-like globin genes in BAC clone Oa_Bb-2L7 [GenBank: AC195438], and two β-like globin genes in BAC clone Oa_Bb-484F22 [GenBank: AC192436 reverse direction].

|  | **Exon 1** | | **Exon 2** | | **Exon 3** | | **Poly-A** |
| --- | --- | --- | --- | --- | --- | --- | --- |
| AC203513 | |  |  |  |  |  |  |
| **ζ** | 3746 | - 3840 | 4178 | - 4382 | 4497 | - 4625 | 4744 |
| **ζ’** | 8545 | - 8639 | 8976 | - 9180 | 9283 | - 9411 | 9544 |
| **α^D^** | 11992 | - 12083 | 13398 | - 13602 | 15213 | - 15341 | 15418 |
| **α^3^** | 20056 | - 20147 | 20553 | - 20757 | 20909 | - 21037 | 21131 |
| **α^2^** | 27074 | - 27168* | 27889 | - 28093 | 28249 | - 28377 | 28492 |
| **α^1^** | 32437 | - 32528 | 32934 | - 33138 | 33290 | - 33418 | 33512 |
| **ω** | 39008 | - 39099 | 39356 | - 39578 | 39690 | - 39818 | 39887 |
| GBY | 48012 | - 47915 | 44550 | - 44328 | 41277 | - 41134 | 40500 |
|  |  |  |  |  |  |  |  |
| AC192436 | |  |  |  |  |  |  |
| **ζ** | 137751 | - 137845 | 138183 | - 138387 | 138502 | - 138630 | 138749 |
| **ζ’** | 142550 | - 142644 | 142981 | - 143185 | 143288 | - 143416 | 143549 |
|  |  |  |  |  |  |  |  |
| AC192436 | |  |  |  |  |  |  |
| **ε** | 79553 | - 79644 | 79788 | - 80010 | 80485 | - 80613 | 80709 |
| **β** | 91660 | - 91751 | 91905 | - 92127 | 92566 | - 92694 | 92765 |
|  |  |  |  |  |  |  |  |

* the α*^2^* gene has two sequencing errors in its exon 1 at positions 2 (A->T) and 7 (G->C), which was confirmed by a BLAST search of the platypus WGS database.
